# Prevalence of insulin resistance and its associated factors in drug-naïve patients with bipolar disorder among Han Chinese population

**DOI:** 10.1186/s12888-024-05838-5

**Published:** 2024-05-23

**Authors:** Kanglai Li, Tong Li, Ting Yang, Youzhen Lin, Yingtao Liao, Zhaoyu Gan

**Affiliations:** Third Affiliated Hospital of Sun Yat-sen University, No. 600 of Tianhe Road, Guangzhou, Guangdong China

**Keywords:** Bipolar disorder, Insulin resistance, Metabolic syndrome, Waist circumference, Clinical characteristics

## Abstract

**Background:**

Metabolic syndrome (Mets) is commonly seen in bipolar disorder (BD). As the key component and early biological index of Mets, insulin resistance (IR) among BD has received more and more attention. However, little is known about the prevalence of IR and its associated factors in drug-naïve patients with (BD), especially among Han Chinese population.

**Methods:**

A cross-sectional study was conducted on 125 drug-naïve patients with bipolar disorder (BD) and 85 healthy controls (HC). The Homeostatic Model Assessment of insulin resistance (HOMA-IR) was calculated, and IR was defined as HOMA-IR greater than the 75th percentile value for health controls (2.35). Clinical characteristics of BD were collected through semi-structural interview performed by a trained interviewer with background of psychiatric education.

**Results:**

Among the measured anthropocentric variables including BMI, waist circumference, abdomen circumference, hipline, and hip-waist ratio, waist circumference was found to be the most closely related to IR (0R = 1.070, 95%CI = 1.031–1.110, *P* < 0.001). Male was another factor that was associated with IR (OR = 2.281, 95%CI = 1.107–4.702, *P* = 0.025). After adjusted for gender and waist circumference, the risk of IR was significantly higher in bipolar disorder than in healthy controls (OR = 2.66, 95%CI = 1.364–5.214, *P* = 0.004). No significant association was found between IR and any of the observed physical and mental comorbidities, any characteristic of illness course including age onset, number of mixed episodes, types of current state, duration of current episode, duration of illness course, rapid cycling, number of mood episodes, and subgroup of BD. Hypersomnia was the only symptomatic feature that was significantly associated with IR (OR = 0.316, 95%CI = 0.124–0.803, *P* = 0.016).

**Conclusions:**

Bipolar disorder increases two-to-three-fold risk of IR, both circumference and male are the risk factors of IR but hypersomnia act as a protective factor.

## Introduction

Compared to general population, patients with bipolar disorder have nearly two-to-three-fold increased risk of type 2 diabetes [1, 2] or metabolic syndrome [3, 4]. In addition to increasing risk of cardiovascular diseases [5] and related death [6], comorbid metabolic dysfunction might worsen the clinical outcome of BD. Cross-sectional studies find that patients with bipolar disorder and type 2 diabetes or insulin resistance have a more chronic course of illness [7, 8], higher odds of rapid cycling [7], worse social function impairment [9], and are more likely to be refractory to mood stabilizer treatment [7, 10]. Longitudinal study also indicates that insulin resistance is associated with a more severe course of bipolar disorder [11]. In addition, among bipolar disorder patients without diabetes mellitus, insulin resistance is negatively associated with memory performance [12]. However, not all the studies find a positive relationship between bipolar disorder and metabolic dysfunction. For example, a cross-sectional study from Brazil finds that insulin resistance in mood disorder is associated with BMI or increased uric acid, instead of mood disorders [13]. Three prior case-control studies do not find a higher prevalence of metabolic syndrome in newly diagnosed bipolar disorder patients than in healthy controls [14–16]. Reasons for this inconsistency between studies might include: the effect of medication is not excluded, and the sample size is too small. It is well recognized that many mood stabilizers, especially the second-generation antipsychotics, could lead to metabolic dysfunction [17, 18]. In the previous studies [1, 3, 4] that reported a higher prevalence of metabolic dysfunction did not exclude patients under medication treatment. While in studies that did not find any difference in insulin resistance between BD and control had a small sample size ranging from 20 to 56, which might be too small to detect any potential significant difference. In addition, when they assessed the association between BD and metabolic dysfunction, they seemed to use analogous but actually different terms, including type 2 diabetes mellitus, metabolic syndrome, impaired glucose metabolism. Although insulin resistance is a key factor in the pathogenesis of diabetes mellitus or impaired glucose metabolism and closely related to metabolic syndrome, IR may not all develop into diabetes mellitus or impaired glucose metabolism or completely overlap with metabolic syndrome [19].

As a key component and early biological index of metabolic syndrome, insulin resistance is centered in a network of physiological interactions, which pulls one node or arm out of the normal state and drags other nodes and arms into the pathological range, including glucose intolerance, overt diabetes, body lipidaemia, hypertension and vascular dysfunction [20]. In the brain, insulin receptors are expressed on all the cells, especially in the olfactory bulb, hypothalamus, hippocampus, cerebral cortex, striatum and cerebellum [21]. Insulin not only provides powerful regulatory control of brain metabolism but also plays a crucial role in learning, neuroplasticity, neuronal cell survival, and other synaptic processes by acting on a host of biological pathways [22, 23]. On the contrary, insulin resistance is thought to be a ‘testable and potentially modifiable risk factor for neuroprogression’ as recently reviewed [5]. Metabolically, bipolar disorder can be viewed as a fluctuation between high energy in mania and low energy states in depression [24]. Therefore, it is reasonable to speculate that IR might play a role in the pathogenesis of BD. Although IR is specifically comorbid with BD [25], it is not a side qua none of BD. Thus, we hypothesis that IR only exist in some specific subpopulation of BD. In this study, we are going to assess the prevalence of IR in drug-naïve patients with bipolar disorder among Han Chinese population and further explore what subpopulation of BD might be associated with IR. As far as we know, no similar study has been done before, especially among Han Chinese population.

## Subjects and methods

### Subjects

The current study is a cross-sectional analysis of a prospective longitudinal single-center study conducted in the Department of Psychiatry and Psychology of the Third Affiliated Hospital of Sun Yat-Sen University. We recruited participants from August 8, 2012, to January 6, 2018. The study was approved by the Clinical Research Ethics Committee of the Third Affiliated Hospital of Sun Yat-Sen University (approval number: [2017]02-233-01). Written informed consents were obtained from all participants. Participants who aged under 18 were required to provide written informed consent from their guardians.

The potential eligible cases were recommended by their first visited psychiatrists to our study team. The healthy controls(HC) were volunteers recruited from the local community during the same period. All the participants were Han Chinese, aged between 16 and 65, had no current active physical illness confirmed by reviewing their previous medical records and routine clinical examination, had no history of psychoactive substance abuse in the past six months. The cases also had to meet the following criteria: (a) fulfill the diagnostic criteria of bipolar disorder of any kind according to the Diagnostic and Statistical Manual of Mental Disorders, Fourth Edition, Text Revision (DSM-IV-TR); (b) had not ever received any psychopharmaceutical treatment before; (c) had no comorbid organic mental disorder. The healthy controls were screened for mental disorders using the Chinese version of the Structured Clinical Interview for the Diagnostic and Statistical Manual of Mental Disorders, Fourth Edition, Text Revision (DSM-IV-TR) Axis 1 Disorders (SCID-I), none patient version. The exclusion criteria for all the participants were any DSM-IV-TR diagnose of a lifetime history of schizophrenia, mental retardation, or organic mental disorder, currently pregnant or breastfeeding, under steroid drugs or other medication treatment which might lead to metabolic dysfunction, or had current active physical diseases confirmed by inquiry of medical history, physical examination, and recent laboratory examination including but not limited to routine blood test, liver and kidney function, thyroid function, abdominal color ultrasound.

### Demographic characteristics

Demographic characteristics, including age, gender, education, marital status, were collected via a questionnaire designed by the researchers.

#### Clinical characteristics

Diagnosis of mental comorbidities was made according to the DSM-IV-TR based on the subjects’ history of present illness and routine mental examination. Physical comorbidities were confirmed by reviewing the patients’ previous medical history and medical records stored in our hospital’s electronic medical system. The characteristics of illness course of BD, including age onset, number of previous depressive/(hypo)manic/mixed episodes, maximum duration of previous depressive/(hypo)manic/mixed episode, and type of current episode were assessed by a semi-structural clinical interview designed by our study team. Rapid cycling was defined as having at least four episode of depression or (hypo)mania per year. Severity of the current episode were evaluated by the 17-item Hamilton Depression Rating Scale (HDRS) [26] and the Young Mania Rating Scale (YMRS) [27]. Physical comorbidities were provided by the participants and confirmed by reviewing the participants’ medical records stored in our hospital’s electronic medical system. Atypical features were assessed by the corresponding section of the Chinese version of SCID-I [28] based on the patients’ most severe depressive episode. Psychotic features were assessed by evaluating whether the participants demonstrated any of the psychotic symptoms, including hallucination, delusion, or disorganized behavior in the course of illness. All the above-mentioned interviews were conducted by the same trained psychiatrist in our research team.

### Metabolic indicators

#### Anthropocentric measures

Anthropocentric measures were conducted by the same nurse, including body weight, height, waist circumference, abdomen circumference, and hipline. Body mass index (BMI) was calculated as weight (kg)/height squared (m^2^). The above-mentioned were measured according to the following protocols [29]:

##### Height

before the measurement of height, the measured participant was required to stand upright with arms loosely to the side, back straight, heels against a vertical measure and the head in the Frankfort plane and put their feet together (both legs were loaded). Height is measured after a deep in-breath, ensuring the head remains in the correct position.

##### Weight

the measured participants were asked to remove shoes, outer garments such as jackets and cardigans, heavy jewelry, loose change, and keys, and then stand with their feet together in the center of the scales with heels against the back edge with arms hanging loosely by their sides and head facing forward, not down.

##### Waist circumference

First, the measured subjects should remove bulky outer or tight garments and shoes with heels, empty their bladder. Then they stand upright with arms loosely to the side. When the waist circumference is measured, the tape is passed round the body and positioned mid-way between the iliac crest and costal margin of the lower rib ensuring it is horizontal and untwisted. At the same time, the measured persons are asked to look ahead and breathe out and the measurement is taken at the end of expiration.

##### Hipline

First, the measured persons should be prepared as for measuring waist circumference. In addition, the subjects should not contract their gluteal muscles before the measurement is taken. When hip circumference is measured, the tape should be passed round the body and positioned at the widest part over the buttocks and below the iliac crest, ensuring it is horizontal and untwisted,.

##### Abdominal circumference

The preparation is the same with that for measuring waist circumference. The tape is applied at the L4-L5 region of the abdomen, midway between the iliac crest and the lowest palpable rib and measurement is taken at the end of normal expiration.

### Laboratory variables

All blood specimens were collected after an overnight fast of at least 10 h and were transported to the clinical laboratory of the Third Affiliated Hospital of Sun Yat-sen University within 3 h。Fasting glucose was measured by the colorimetric method, and.

serum insulin was measured by electrochemiluminescence immunoassay. The Homeostatic Model Assessment of insulin resistance (HOMA-IR) was calculated as follows [30, 31]: fasting plasma glucose (mmol/L) ×fasting serum insulin (µU/mL)/22.5. Considering the HOMA-IR cut-off points to diagnose insulin resistance vary from race to race, IR here is defined as a value greater than the 75th percentile value for health controls (2.35) according to the World Health Organization (WHO) [32].

### Statistical analyses

All statistical analyses were performed using SPSS 22.0 (SPSS Inc, Chicago, IL, USA). Continuous data was reported as median with interquartile ranges for non-normally distributed variables or mean ± SD for normally distributed variables, while categorical data was reported as n (%). The Shapiro-Wilk test was used to test for normality distribution of continuous data. Differences between groups were compared using a chi-square test for categorical data and using a student t test or a Mann-Whitney U test for continuous data. Potential confounding factors that might be associated with IR were selected by multivariate binary logistic regression with IR as dependent variable and all the demographic and anthropocentric features of participants as independent variables (method = forward stepwise, likelihood ratio). Then, univariate logistic regression was performed to further examine each clinical feature by adjusting for potential confounding factors. The result was considered significant at *P* < 0.05 and corrected for multiple testing using a Bonferroni correction. However, the un-corrected *p*-value is still presented when the Bonferroni-corrected threshold for statistical significance is reported with each table because only showing corrected value, important findings may be missed. Odds ratios and 95% confidence intervals were used to quantify the strength of associations.

## Results

### Comparison of demographic and physical characteristics between BD and HC

The demographic and anthropocentric features of all participants are showed in Table 1. Total of 210 participants were recruited in this study, including 125 patients with BD and 85 HC. No significance was found in age and marital status between BD groups and HC group. However, the educational level of BD group was lower than that of HC group (12.7 ± 3.1 VS. 16.6 ± 3.0, *P* < 0.001), while the proportion of female subjects among BD group was also lower than HC group (55.2% VS. 76.5%).

As for the anthropocentric features, no significant difference was found between BD and HC group in BMI and waist circumference, but the abdominal circumference, hipline and the waist-to-hip ratio were all significantly larger in BD group than in HC group (all *P* < 0.05).

**Table 1:** Comparison of demographic, physical characteristics, HOMA-IR and the prevalence of IR between bipolar disorder and health controls

	Bipolar disorder *N* = 125	Control *N* = 85	*P*
Age (mean ± SD) (year)	27.9 ± 11.0	29.5 ± 11.7	0.303
Education (mean ± SD) (year)	12.7 ± 3.1	16.6 ± 3.0	**< 0.001**
Female n(%)	69(55.2)	65(76.5)	**0.02**
Married n(%)	39(31.2)	21(24.7)	0.217
BMI	21.5 ± 3.0	21.3 ± 3.9	0.711
Abdominal circumference (mean ± SD) (cm)	79.8 ± 9.4	76.4 ± 9.2	**0.011**
Waistline (mean ± SD) (cm)	74.9 ± 9.3	75.2 ± 9.8	0.778
Hipline (mean ± SD) (cm)	91.6 ± 8.4	87.4 ± 10.1	**0.001**
waist-to-hip ratio	0.87 ± 0.13	0.82 ± 0.07	**0.001**

### Comparison of fasting glucose, fasting insulin, HOMA-IR and IR between BD and HC

First, we compared the fasting glucose, fasting insulin, and HOMA-IR between BD group and HC group. As shown in Table 2, both fasting glucose (4.82 ± 0.81 VS. 4.52 ± 0.55, *P* = 0.03) and fasting insulin [9.55(6.83–11.51) VS. 7.95(5.64–11.68), *P* = 0.013] were significantly higher in BD than in HC. At the same time, HOMA-IR in BD [1.99(1.42–3.05)] were also significantly higher than that in HC [1.59(1.14–2.35)] (*P* = 0.002). The prevalence of IR in BD group (40.8%) and HC group (24.7%) were calculated and listed in Table 2. Chi-square test showed a significant higher prevalence of IR in BD group than in HC group (*P* = 0.011). Considering BD group and HC group did not match with each other in gender proportion and some anthropocentric features, binary logistic regression model was built with IR (1 represent having IR, 0 represents no IR) as dependent variable, gender, education, age, BMI, abdominal circumference, waist circumference, hipline, waist-to-hip ratio and group (1 = BD, 0 = HC) as independent variables (method = forward stepwise, likelihood ratio). According to the result listed in Table 3, gender, waist circumference and bipolar disorder remained in the equation. Among the measured anthropocentric variables including BMI, waist circumference, abdomen circumference, hipline, and hip-waist ratio, waist circumference was found to be the most closely related to IR (OR = 1.070, 95%CI = 1.031–1.110, *P* < 0.001). Gender was another factor that was associated with IR (OR = 2.281, 95%CI = 1.107–4.702) (female was chosen as the reference). After adjusted for gender and waistline, the risk of IR was significantly higher in BD group than in HC group (OR = 2.66, 95%CI = 1.364–5.214, *P* = 0.004).

**Table 2:** Comparison of fasting glucose, insulin, HOMA-IR and the prevalence of IR between bipolar disorder and health controls

	Bipolar disorder *N* = 125	Control *N* = 85	*P*
Fasting glucose (mean ± SD) (mmol/L)	4.82 ± 0.81	4.52 ± 0.55	**0.03**
Fasting insulin (mean ± SD) (mU/L)	9.55(6.83–11.51)	7.95(5.64–11.68)	**0.013**
HOMA-IR median (25-75%)	1.99(1.42–3.05)	1.59(1.14–2.35)	**0.002**
IR (%)	40.8%	24.7%	**0.011**

**Table 3:** Results of logistic regression of demographic and physical characteristics and group on insulin resistance

	Wald	*P*	OR (95%CI)
Gender	6.482	0.025	2.281(1.107–4.702)^a^
Waistline	17.437	< 0.001	1.070(1.031–1.110)
Group^a^	5.176	0.004	2.667(1.364–5.214)^b^

Note: a. female was chosen as the reference

b. health control group was defined as the reference

### The association between comorbidities and insulin resistance

We looked next at the association between comorbidities and insulin resistance among BD. We regressed total and each specific comorbidity on the prevalence of IR, all adjusting for waistline and gender since they were the major confounding factors according to the above-mentioned statistical analyses. It came out that none of the observed physical or mental comorbidity, no matter as a group or a specific one, was significantly associated with IR (all *P* < 0.05), seen in Table 4.

**Table 4:** The association between comorbidities and insulin resistance among BD

	*n*(%)	*P* ^i^	OR(95%CI)
**Physical comorbidities (total)**	53(42.4)	0.693	1.117(0.645–1.934)
Inflammatory or Autoimmune diseases^a^	26(20.8)	0.799	1.133(0.432–2.972)
Cyst or hyperplasia^b^	20(16.0)	0.479	0.677(0.229–1.997)
Thyroid diseases^c^	7(5.6)	0.198	3.279(0.538–19.975)
Glycolipid metabolic diseases^d^	5(4.0)	0.963	1.048(0.104–7.844)
Cardiovascular diseases^e^	9(7.2)	0.679	1.349(0.327–5.562)
**Mental comorbidities (total)**	36(28.8)	0.549	0.782(0.350–1.748)
OCD	7(5.6)	0.438	0.495(0.084–2.932)
Anxiety disorders^f^	6(4.8)	0.822	0.811(0.131–5.030)
Eating disorders^g^	3 (2.4)	0.577	2.144(0.147–31.242)
Somatoform disorders	9(7.2)	0.460	1.732(0.404–7.430)
Attempted suicide	3 (2.4)	0.999	0.000(0.000-)
Psychoactive substance abuse^h^	10 (8.0)	0.793	0.817 (0.182–3.681)

Note:

a. Including all kinds of asymptomatic infectious diseases, allergic rhinitis, urticaria, eczema, psora, asthma, and irritable bowel syndrome.

b. Including all the benign cyst, nodule, polyp and well-healed cancer.

c. Including Grave’s diseases, thyroiditis, thyroid cyst, thyroid nodule, thyroid cancer, primary hypothyroidism.

d. Including diabetes mellitus, impaired glucose tolerance, and hepatic adipose infiltration.

e. Including hypertension, lacunar cerebral infarction, aneurism, phlebangioma, atherosclerosis, and varicosity.

f. Including panic disorder, generalized anxiety disorder, and social phobia.

g. Including bulimia nervosa and anorexia nervosa.

h. Including 7 with smoking, 2 with alcohol drinking, 1 with smoking and alcohol drinking.

i. Adjusting for gender and waistline. *p* < 0.05/13 = 0.0038 (required significance level after Bonferroni correction for 13 multiple testing).

### Association between characteristics of illness course and insulin resistance

Subsequently, we examined the relationship between characteristics of illness course of BD and IR. As demonstrated in Table 5, when number of mood episode of any kind was treated as a continuous variable, none of them was significantly associated with insulin IR (all *P* > 0.05). Other characteristics of illness course, including age onset, number of mixed episodes, types of current state, duration of current episode, duration of illness course, rapid cycling, and subgroup of BD did not show any significant relationship with IR (all *P* > 0.05).

**Table 5:** The association between characteristics of illness course of BD and insulin resistance

items	Characteristics of illness course	*P* ^a^	OR(95%CI)
Onset age (mean ± SD) (year)	24.4 ± 10.6	0.803	1.005(0.967–1.044)
Number of depressive episodes Median (25-75%)	1(0–2)	0.068	0.790(0.613–1.018)
Number of (hypo)manic episodes Median (25-75%)	1(0–1)	0.158	0.827(0.636–1.076)
Number of mixed episode Median (25-75%)	0(0–1)	0.248	0.647(0.310–1.353)
Number of mood episodes Median (25-75%)	2(2–4)	0.072	0.855(0.721–1.014)
Current state			
Depressive	74(59.2)		reference
(hpyo)manic	13(10.4)	0.467	1.729(0.396–7.555)
mixed	38(30.4)	0.748	1.172(0.444–3.091)
Duration of current episode (25-75%) (day)	100(30–365)	0.426	1.00(0.998–1.001)
Duration of illness Median ( 25-75%) (year)	2(1–2)	0.429	0.951(0.840–1.153)
Rapid cycling n(%)	19(9.0)	0.114	0.375(0.111–1.264)
Subgroup of BD			
NOS n(%)	10(0.08)		Reference
BPI n(%)	21(16.8)	0.486	1.885(0.317–11.211)
BPII n(%)	66(52.8)	0.565	1.586(0.330–7.632)
BP(mixed) n(%)	28(22.4)	0.773	1.280(0.240–6.834)

Note: a. adjusting for gender and waistline, *p* < 0.05/13 = 0.0038 (required significance level after Bonferroni correction for 13 multiple testing)

### Association between symptomatic features of BD and insulin resistance

Finally, we explored the relationship between symptomatic features of BD and IR. According to the result listed in Table 6, only hypersomnia was found to be significantly associated with IR (OR = 0.316, 95%CI = 0.124–0.803, *P* = 0.016), while other symptomatic features including total scores of HAMD-17 and YMRS, other atypical symptoms of depressive episode, and psychotic feature were not significantly related to IR (all *P* > 0.05).

**Table 6:** The impact of symptomatic features on the insulin resistance of BD

items	Symptomatic features	*P* ^a^	OR(95%CI)
HAMD-17 total scores (mean ± SD)	20.6 ± 8.1	0.530	0.984(0.937–1.034)
YMRS total scores (mean ± SD)	10.4 ± 10.7	0.724	0.993(0.955–1.032)
Atypical feature			
Mood reactivity n(%)	84(67.2)	0.203	0.581(0.252–1.341)
Increased appetite n(%)	32(25.6)	0.849	1.091(0.445–2.675)
Weight gain n(%)	25(20.0)	0.397	0.648(0.238–1.767)
Hypersomnia n(%)	39(31.2)	**0.016**	**0.316(0.124–0.803)**
Leaden paralysis n(%)	45(36.0)	0.956	0.977(0.432–2.211)
Reject sensitivity n(%)	70(56.0)	0.360	1.456(0.652–3.251)
Psychotic feature n(%)	26(20.8)	0.250	0.548(0.197–1.527)

Note: a. adjusting for gender and waistline, *p* < 0.05/9 = 0.0056 (required significance level after Bonferroni correction for 9 multiple testing)

## Discussion

The objective of this study was to assess the prevalence of IR among drug-naive patients with BD and to examine whether IR was associated with specific clinical characteristics. All the participants were patients who visit psychiatrists for the first time and completed the measurement of metabolic function before the initiation of psycho-pharmaceutical treatment. Therefore, the results could reflect the true relationship between IR and BD.

In this sample, the risk of IR among BD was 2.66 times higher than among HC, in line with previous reports suggesting that type 2 diabetes [1] or metabolic syndrome [3, 4]occur two to three times as often in patients with BD as compared with general population. After adjusting for potential confounding factors, male population showed more than two times higher risk of IR than female population, which is consistent with the conclusion from a previous review that women have a more insulin-sensitive environment than men [33]. The gender difference in insulin-sensitivity might partly be attributed to differences in sexual hormones [34], adipokines including leptin and adiponectin [33], and coping strategies for stress [35] between women and men.

Our study found that waist circumference was the most closely related factor to IR among several anthropocentric variables including BMI, waist circumference, abdomen circumference, hipline, and hip-waist ratio, duplicating the view from previous study that waist circumference is an essential factor in predicting IR [36]. Possible explanation for this is that waist circumference is the best marker of metabolic risk of visceral fat [37], while visceral abdominal fat is the best predictor of IR [38].

We did not find any significant association between any physical or mental comorbidity with BD and IR after adjusting for gender and waist circumference. However, the following information should be noticed while interpreting this finding. First, the comorbidities in a specific group might not be homogeneous in terms of insulin sensitivity. For example, in diabetes mellitus, type 1 diabetes is accompanied by insulin resistance while type 2 diabetes exhibits self-reactivity [39], so dose eating disorder. Anorexia nervosa demonstrates increased insulin sensitivity whilst bulimia nervosa and binge-eating disorders show decreased insulin sensitivity [40]. Second, the rate of some comorbidity might be too low to detect any significant difference between comorbid group and non-comorbid group. Third, all the comorbid physical diseases are in stable period and do not activate the immune system and cause inflammation, and the latter is thought to play an important role in inducing IR [41]. Finally, the accumulation of fat depot in visceral fat is believed to contribute to development of IR, the reported positive relationship between some mental disorder like OCD and IR or metabolic syndrome might be mediated by the accumulation of visceral fat [42]. However, once adjusted for waist circumference, the best marker of metabolic risk of visceral fat, just as we did, the relationship between the comorbid conditions and IR might disappear.

Contrary to the findings from previous studies that compared to euglycemic patients with BD, BD patients with impaired glucose metabolism (IGM) (including IR, glucose intolerance, and diabetic mellitus) were younger [7], had higher rate of rapid cycling [26]and chronic course [7, 9], longer illness duration [8], higher number of previous manic/hypomanic episode, and higher ratio of manic/hypomanic to depressive episodes [8], our study found no significant association between IR and any characteristics of illness course of BD, including onset age, duration of illness, length, severity and type of current episode, number of mood episodes and rapid cycling. One possible explanation for this difference might be: in studies reporting a positive association between number of episodes or duration of illness and IR, patients with higher number of episodes or other clinical characteristics, are exposed to psychopharmaceutical treatment longer or more heavily, therefore more likely to trigger treatment-inducing IR. Although in some study, pharmaceutical treatment is adjusted in the process of statistical analysis, the effect of adjustment is limited since the impact of treatment on IR is highly different between individuals and medications [18]. However, in our study, all the participants are drug-naïve, so the impact of pharmaceutical is excluded and remains homogenous between groups. In addition, compared to the above-mentioned studies, our study had a younger population, less comorbid rate of physical diseases and all the participants were Han Chinese. These differences might also contribute to the above inconsistency between studies.

Finally, we observed a negative relationship between hypersomnia and IR independent of waist circumference. As far as we know, no study has ever examined this before among BD population. However, in patients with depression from western countries, hypersomnia and hyperphagia were found to be positively associated with HOMA-IR or level of insulin [43, 44]. BMI, C-reactive protein, and race were reported to mediated the association between hyperphagia or hypersomnia and insulin resistance [43, 45]. Therefore, it is speculated that difference in ethnicity and BMI might contribute to the different association between atypical features of depressive episode and IR.

Our findings should be considered in the context of the following limitations: First, patients were recruited because they sought medical help in our hospital while the healthy controls were selected not randomly from social community. Therefore, potential selection bias might exist, and the cut-off point of IR might be less representative of the general Han Chinese population. Second, the strict exclusion criteria of this study might guarantee a sample of high homogeneity but might make the sample less representative. Third, the cross-sectional design of this study could not infer any causal relationship between IR and any clinical characteristic of BD. Forth, the characteristics of illness course of BD and symptomatic features of depressive episodes were collected retrospectively, so recall bias could not exclude. Fifth, the sample size for some clinical characteristics might be too small to detect potential difference.

In sum, our study finds that the prevalence of IR is more than two times higher among drug-naive patients with BD than healthy control among Han Chinese population. Male and waist circumference are all the risk factors of IR. These findings might help guide the prevention and treatment of metabolic syndrome in BD patients among Han Chinese population, but further study is needed to explore how these clinical characteristics relate with IR.

## Data Availability

The original data is kept by the corresponding author to prevent information leakage. If necessary, the original data can be requested from the corresponding author on reasonable request.
